# Learning from a Rapid Health Impact Assessment of a proposed maternity service reconfiguration in the English NHS

**DOI:** 10.1186/1471-2458-8-138

**Published:** 2008-04-25

**Authors:** Greg Fell, Sophie Haroon

**Affiliations:** 1 Yorkshire and Humber Public Health Training Programme, Yorkshire Deanery, c/o Academic Unit of Public Health, Institute of Health Sciences, University of Leeds, 101 Clarendon Road, Leeds, LS2 9LJ, UK

## Abstract

**Background:**

Within many parts of the country, the NHS is undertaking reconfiguration of services. Such proposals can prove a tipping point and provoke public protest, often with significant involvement of local and national politicians. We undertook a rapid Health Impact Assessment (HIA) of a proposed reconfiguration of maternity services in Huddersfield and Halifax in England. The aim of the HIA was to help the PCT Boards to assess the reconfiguration's possible consequences on access to maternity services, and maternal and infant health outcomes across different socio-economic groups in Kirklees. We report on the findings of the HIA and the usefulness of the process to decision making.

**Methods:**

This HIA used routine maternity data for 2004–2005 in Huddersfield, in addition to published evidence. Standard HIA techniques were used.

**Results:**

We re-highlighted the socio economic differences in smoking status at booking and quitting during pregnancy. We focused on the key concerns of the public, that of adverse obstetric events on a Midwife Led Unit (MLU) with distant obstetric cover. We estimate that twenty percent of women giving birth in a MLU may require urgent transfer to obstetric care during labour. There were no significant socio economic differences. Much of the risk can be mitigated though robust risk management policies. Additional travelling distances and costs could affect lower socio-economic groups the greatest because of lower car ownership and geographical location in relation to the units. There is potential that with improved community antenatal and post natal care, population outcomes could improve significantly, the available evidence supports this view.

**Conclusion:**

Available evidence suggests that maternity reconfiguration towards enhanced community care could have many potential benefits but carries risk. Investment is needed to realise the former and mitigate the latter.

The usefulness of this Health Impact Assessment may have been impeded by its timing, and the politically charged environment of the proposals. Nonetheless, the methods used are readily applicable to assess the impact of other service reconfigurations. The analysis was simple, not time intensive and used routinely available data. Careful consideration should be given to both the timing and the political context in which an analysis is undertaken.

## Background

Within many parts of the country, the NHS is undertaking reconfiguration of services. The push for this is coming from a number of sources; the drive for greater efficiency, choice, practical issues such as workforce shortages and the Working Time Directive [1]. In many districts, commissioners and provider organisations are initiating significant consultations about altering the configuration of services provided locally. Such proposals can prove a tipping point and provoke public protest on a large scale, often with significant involvement of local and national politicians [2].

The maternity care aspect of one such planned reconfiguration [3] set out a proposal to close the Obstetric led unit at Huddersfield and centralise this service at a site 5 miles more distant (Halifax), leaving a Midwife Led Unit in Huddersfield hospital. Concurrently savings released from the reconfiguration of hospital services were to be invested in community ante natal services, particularly targeted in more deprived areas. The desired outcome of this proposed maternity service reconfiguration was to enable an improvement in ante natal care; consequently improving the management of risk more proactively. Concurrently the establishment of a Midwife Led Unit in Huddersfield would enable many women the choice of whether to give birth without obstetric intervention whilst maintaining obstetric cover in another hospital that was part of the same NHS Trust.

The Hospital Trust concerned provided the principal district general hospital service for Huddersfield town, the surrounding area to the South and West, and Halifax Town and surrounding areas. There are two hospital sites within the trust, one in Huddersfield and one in Halifax. The straight line distance between sites is approximately 5 miles. The Trust falls within the Kirklees and Calderdale local authority districts. In total the catchment area is approximately 418,000 people. The main commissioning body for the Hospital Trust is Calderdale Primary Care Trust (PCT), and the former South and Central Huddersfield (now part of Kirklees) PCT. The social and demographic characteristics of the area are similar to other parts of the UK [4]. The absolute numbers of births in each hospital is similar; approx 2,500.

The public consultation on this proposed reconfiguration provoked strong local protest from health care professionals, residents and local politicians [5,6], chiefly focused on the risks associated with emergency transfers of labouring women between hospital sites. The reconfiguration plan set out that any resource savings released from the changes to hospital services were to be reinvested in community ante natal services.

We were commissioned by the Director of Public Health of one of the PCTs concerned to undertake a health impact assessment to give an indication of the potential population health impact of this proposal. The aim of the HIA was to help the PCT and Hospital Trust Boards to assess the reconfiguration's possible consequences on access to maternity services, and maternal and infant health outcomes across different socio-economic groups in Kirklees.

The timing of the conduct of this health impact assessment was towards the end of the period of consultation and was timed to be complete close to the time of a joint PCT/Hospital Trust board meeting to discuss support for the proposed reconfigurations. There was no a priori assurance that any of the decision making bodies would be willing to act on our results; our review was intended to *inform *the decision making process.

This health impact assessment took place in 2006, i.e. prior to the 2007/08 NHS Operating Framework and before Maternity Matters ^1 ^(2007) report was published. What we present is an example of a health impact assessment undertaken rapidly; with minimal national guidance and done in a politically charged atmosphere. We offer an example of how HIA could be done within the limits of the practical day-to-day working a PCT; including our reflections on the process and advice to those undertaking similar work. We were not aware of any other health impact assessments that had been undertaken in this area; to our knowledge this was the first piece of work in this area. Maternity Matters highlights health impact assessments as an important tool and to be included in service reviews currently undertaken by PCTs^1^.

## Methods

Significant stakeholder involvement had already been undertaken so we conducted the assessment as a desktop exercise using routinely available data and published literature; using the framework for HIA set out by the National Institute of Clinical and Health Excellence [7].

We used a one year cohort of Hospital Episode Statistics for women giving birth in 2004. We also conducted a review of published literature around maternity service configuration and impact on outcomes. We used both data sources to make a judgement about possible antenatal, intrapartum and postnatal outcomes of the proposed reconfiguration, as was described in the consultation documents. We particularly focused on health inequalities.

## Results

A copy of the final report is available [8]. Brief results are presented here.

### Ante Natal Care

We used smoking status as an indicator for infant health of antenatal care. There were significant socio-economic variations in smoking prevalence at booking and at delivery. Many women did quit, however there was no statistically significant difference, between different socio economic groups, of the percentage of smokers quitting during pregnancy.

### Intra-partum care

The key concerns of the public, politicians and some healthcare professionals revolved around the increase in the potential for adverse events associated with the proposed Midwife Led Unit in Huddersfield, **loss of local hospital with doctor-cover (in Huddersfield), more travel and inconvenience. **Concerns centred on the safety of stand alone MLUs, no immediate obstetric cover, and the safety of transferring a woman to the obstetric-led unit at CRH should she develop problems intrapartum. It seemed apparent that the public were less aware of other aspect of the reconfiguration; such as proposed improvements in ante natal care.

To attempt to quantify the potential number of complications on the MLU in Huddersfield, we conducted an analysis of births using routinely available data, over a one year. Most of the 2,543 births at HRI were normal vaginal deliveries without complications. There was no statistically significant socio economic difference in deliveries that required instrumental support or experienced complications.

We hypothesised that certain events intrapartum, should they occur on the proposed MLU in Huddersfield would require obstetric intervention (and thus urgent transfer to the obstetric-led unit in Halifax). We used primary diagnosis codes (ICD10) for key complicating factors of labour; and analysed their frequency and social spread. We excluded a number of factors that would most likely have been picked up antenatally (e.g. pre-existent hypertension, pre-eclampsia, damage to fetus from alcohol/drug use, previous ante-partum haemorrhage, and previous preterm delivery). With enhanced antenatal care, these women should be identified antenatally; the pregnancy classed as 'high-risk' and advised not to deliver at the MLU.

Thus, we focussed our analysis on four main complications (shoulder dystocia, fetal distress, cord prolapse and obstructed labour) – that could arise during labour and are not easily foreseeable. From a one year cohort of all births at HRI, we found 511 (or approximately 20% of all births) episodes of these major complicating factors that would have required a woman to be urgently transferred to the care of an obstetrician (Table 1). There was no apparent socio-economic difference in complications between the affluent and deprived groups. Thus 20% of all deliveries would have required (assuming women under the care of a midwife in a MLU) emergency transfer to obstetric care.

**Table 1:** Analysis of annual numbers of potentially unforeseeable intrapartum complications in the Huddersfield birth cohort by socio-economic group

**Major unforeseeable complication**	**Affluent**	**Better Off**	**Average**	**Poor**	**Deprived**	**Total**	**% of total deliveries (2543)**
Shoulder dystocia	0	2	2	4	3	11	0.4
Fetal distress	57	40	69	60	151	380	14.9
Cord prolapse	0	1		1	2	4	0.2
Obstructed labour	26	12	24	19	35	116	4.6
**Grand total**	**83**	**55**	**95**	**84**	**191**	**511**	**20.1**
**Total births**	**406**	**299**	**497**	**360**	**952**	**2543**	
**% by socio-economic group**	**20.4**	**18.4**	**19.1**	**23.3**	**20.1**	**20.1**	

It is worth noting that there might be other complications of delivery that might require urgent medical attention, these include postpartum haemorrhage (PPH) and neonatal resuscitation. PPH and neonatal resuscitation were not considered in the assessment because the HIA's primary objective was concerned with analysing and producing an impartial view regarding the impact on direct and immediate consequences of labour and intrapartum transfer on the mother between maternity units.

The reconfiguration proposals ^3 ^estimated that about 20% of Huddersfield women would choose to give birth in the MLU in Huddersfield (the remainder travelling to the obstetric unit at Halifax). From this, we estimated that approximately 100 (or one fifth of 511) unforeseeable maternal complications could occur intrapartum on the MLU and require urgent transfer to the obstetric-led unit at CRH. This potential number is consistent with the (then) reported transfer rate between existing midwife care and obstetrician in Calderdale Royal Hospital.

With clear selection criteria of only low-risk mothers to give birth at the proposed MLU and enhanced community provision to mitigate against some of the factors which factor into a poorer labour, we estimate that it is likely that one to two women per week would be transferred; or 2–4% of the total births in Huddersfield per year with no significant socio economic differences.

Concerns had been raised about the transfer time in such instances of unforeseen complication. Data from the local Ambulance Trust [9] suggests that the time between the two hospital sites for a blue light transfer between HRI and CRH is 10–27 minutes. We hypothesised that, for the one to two women per week requiring urgent transfer, there could be potential time to make that transfer without compromising maternal and infant care. Given the gravity of this scenario, this hypothesis should be very carefully tested.

### Postnatal care

If a woman is assessed as having a "high risk" pregnancy and needing obstetric supervision, her only option under the reconfiguration would be to deliver in Halifax. This would incur an increased distance to travel to give birth, a one-off for the woman. However, for visiting relatives we considered the impact of the increased distance, time and cost to travel to the only available obstetric-led unit under the reconfiguration across socio-economic groups. Under a number of assumptions about the number of relative visits, median hospital stay and car ownership levels by socio economic group, we estimated that the burden of additional travel for visiting relatives (to a more distant site) would be greater in the most socially deprived groups. As a proportion of total income, it is more expensive for relatives from deprived areas to visit. Additionally, we estimate a minimum of an additional 165,000 km would be travelled (to a more distant site) by visiting relatives.

We considered breastfeeding rates as an indicator of post natal support (Table 2). As expected there were obvious socio economic differences in infant feeding. Greater community postnatal support (as proposed in the reconfiguration) may lead to the increased continuation of breastfeeding by all socio-economic groups thus contributing to a range of public health outcomes.

**Table 2:** Method of infant feeding at discharge by socio-economic group, for one year's birth cohort in Huddersfield

Socio-economic group	% Breastfeeding (including mixed)	% Bottle feeding	Feeding status unknown
Affluent	69.7	15.1	15.1
Better off	68.6	12.1	19.3
Average	63.8	22.1	14.1
Poor	58.7	30.7	10.7
Deprived	52.9	34.4	12.7

## Discussion

### Limitations of the study

Our simple study had a number of important limitations. The design was simplistic in terms of fully answering the question; we were limited by available time and resources. We used one year's worth of birth data so could not illustrate long-term trends. Some important antenatal and postnatal care data (e.g. gestation at booking, postnatal depression) was not available. The transport findings are limited by our assumptions and need testing.

A number of events (for example PPH or neonatal resuscitation) were not considered in the analysis. Our rationale for this was a perceived need to focus on the key concerns expressed, intrapartum emergency transfer. Also, the extent of neonatal morbidity precipitated by adverse intrapartum effects can be highly variable and is difficult to quantify. Thus within the limits of time allowed for this HIA, only direct and immediate intrapartum maternal events were hence considered.

We concluded that there are risks and benefits associated with this type of reconfiguration. On balance, our view was that the reconfiguration *could *have a number of beneficial effects on addressing health inequalities across the socio-economic groups.

### Ante natal care

It has been suggested by many that socio economic distributions in antenatal risk factors may result in an inequitable distribution of infant health outcomes [10,11]. Poor antenatal attendees include women who are young [12], single, of grand multiparity, and from ethnic minorities [13,14]. Teenage mothers typically have lower birth weight babies, higher infant mortality, higher risk of babies with congenital abnormalities, and are less likely to breastfeed. Poor nutrition and smoking leads to babies with lower birth weights and prematurity. Such factors are more prevalent in disadvantaged areas and thus babies born to mothers in these areas are at highest risk of poor health [15,16].

Improved antenatal care proposed by the reconfiguration could improve ante natal risk and co morbidity management; and reduction in risk. If appropriately targeted to high risk group, and fully implemented, this might also contribute to reductions in inequalities of maternal and neonatal outcomes. However, this improvement in outcomes absolutely depends on improved capacity in and delivery of community antenatal services; which in turn depend on the release of funds to increase capacity. The reconfiguration proposals set this out; it needs to be borne to fruition. Current government policy has acknowledged the importance of a life course approach [17], beginning in the pre-conception period, to reducing inequalities. Within this there is a need to focus on the very early years and support, increasing social support, increasing social capital and coping mechanisms; all of which might act as a buffer against poverty per se. Better antenatal care by itself might not solve poverty; but a multi factorial, multi agency approach might protect the most vulnerable populations against the effects of poverty

Addressing these antenatal risk factors cannot be adequately achieved through a hospital model so the reconfiguration offers hope of benefit. Our HIA highlighted the need to consider the design; type and skill mix of staff providing this enhanced antenatal and postnatal care, particularly with respect to inequalities in outcome. Community ante natal services might not just be delivered by midwives and clinically focused; it might be beneficial for a consideration of a wider range of provision, including health care, health behaviour change, welfare benefits, housing, social support. For example, health trainers and peer support roles might be developed in addition to greater numbers of community midwives. More robust links to other agencies such as housing and welfare rights will need to be created. Additional support must be focused in the most disadvantaged areas to have a meaningful impact on equity. If all this is truly delivered, then the reconfiguration *could *achieve a large impact on improving maternal and neonatal health outcomes, and addressing the inequalities across the different socio-economic groups in Kirklees.

It is possible that with a rapid discharge policy community postnatal care may need to be enhanced, particularly for vulnerable groups. Enhanced postnatal care would facilitate more support of mothers who are young, single, from ethnic minorities and disadvantaged groups. However we stress that these benefits are dependant on additional capacity in community ante and post natal care services.

### Intrapartum care

Our analysis did, however, find some potential problems with the reconfiguration such as the clinical risks associated with the MLU. It was difficult to accurately quantify the additional absolute risk but, on the balance of probabilities, we found that it is likely to be small at the individual level and might be mitigated by careful patient selection and robust patient transfer policies.

With respect to the key risk identified – the safety of midwife led units expressed in terms of perinatal and maternal outcomes, and the risks associated with emergency transfer – there is a dearth of high quality studies upon which to draw firm conclusions [18-20]. Some have suggested that for carefully selected, low-risk patient groups they are as safe as, if not better than obstetric-led units, resulting in lower perinatal and infant mortality, shorter deliveries and fewer interventions [21-23]. We did not consider a number of other potential obstetric/paediatric emergencies, for example major post partum haemorrhage or the need for neonatal resuscitation. Any plan to reduce clinical risk should systematically identify all conceivable risks and implement policies to reduce them. Intrapartum transfer is infrequent; our estimations are consistent with the Department of Health Guidance [24]. Additionally, the risks can be further reduced if strict criteria on the type of woman allowed to give birth in an MLU are adhered to as well as strict criteria for transfer. Evidence suggests patient satisfaction with MLUs is high [25]. This may not be an issue that will be settled by evidence alone; although more evidence will be helpful. There are also conflicting philosophies, ideologies to contend with in addition to very careful risk communication to the public.

The risks associated with emergency transfer are critical, and are perceived by the clinicians involved as the crucial issue in this reconfiguration. Further assurance may be required that the risks identified in Table 1 (and possibly others) can be satisfactorily handled within the transfer time between the two sites. This may require further audit of previous emergency transfers, high levels of training and vigilance by those staffing the Midwife Led Unit.

Despite some limitations (dictated by the speed with which the analysis was required), our analysis addresses some of the important issues related to the reconfiguration of maternity services.

Additional visitor transport time, costs and distances to the obstetric-led unit at CRH were found to disproportionately impact on those from disadvantaged areas. This could potentially worsen inequalities across socio-economic groups but could be mitigated by innovative transport schemes.

We were able to identify potential strategies to mitigate potentially adverse consequences of this reconfiguration (Table 3), and auditable factors for the monitoring of the reconfiguration's impact (Table 4).

**Table 3:** Possible solutions to mitigate potentially adverse consequences of the reconfiguration

**Antenatal care**	Clear targeted investment of ante natal care in the most deprived areas. Links to Children's Centres and other agencies. Investment in alternative models of care, e.g. peer educators, health trainers, in addition to community midwives.
**Hospital and labour care**	Robust supervision of midwives on the MLU. Strict protocols for identification of high-risk pregnancies antenatally and advice to mothers not to use the MLU. Strict protocols for rapid identification of labour complications and rapid transfer. MLU midwives to be trained in neonatal life support. Resuscitation equipment available on the MLU. Telemedicine links with the obstetric-led unit.
**Transport**	Implementation of a range of travel support schemes particularly targeted to low-income groups, e.g. improved bus routes, taxi vouchers, national travel tokens, free shuttle service.
**Sustainability of MLU**	Ongoing hospital trust board support for the MLU and development of skills and capacity within it.

**Table 4:** Auditable suggestions for ongoing monitoring of the reconfiguration's impact

**Antenatal care**	Skills mix and range of staff in additional antenatal care. Antenatal risk management. Inequalities in access and outcome – particularly gestation at booking, smoking status, high-risk pregnancy management, access by minority ethnic groups and teenage mothers.
**Hospital care**	Midwifery resuscitation skills. Use of resuscitation equipment at the Huddersfield MLU. Perineal tears, episiotomies, significant blood loss or problems after delivery, length of labour at Huddersfield MLU. Transfer rates from HRI to CRH. Do they match the estimated 1–2 women per week? Transfer times. Mobilisation times of ambulance crews from the time of being alerted to the time they arrive at the MLU door. Patient satisfaction of community services and MLU.
**Transport**	Uptake of transport and travel schemes by postcode and ethnicity.
**Post natal**	Breastfeeding initiation and continuation. Infant mortality. Postnatal depression rates.

### Conduct of the HIA

This simple study demonstrates that, albeit simple, Health Impact Assessments can be conducted rapidly, cheaply and in a politically charged atmosphere. We offer our tips to others (Table 5). We estimate that the total resource for this work was 1.5 whole time Specialist Registrars in Public Health, for approximately 3 months. We used routinely available data; published literature; the analysis was relatively simple. This methodology is readily applicable to other service reconfigurations.

**Table 5:** 

**Tips for others undertaking HIA**
• HIA can be done quickly with simple analysis.
• Use literature to make informed judgements where there is limited local data.
• Get to the nub of key concerns from stakeholders quickly, particularly where there is opposition. Focus your analysis here, but bear in mind that even the best quality analysis might not overcome trenchant or philosophical opposition to a policy proposal.
• Timing – ensure the analysis is complete in good time to influence or inform decision making. There is a balance between early analysis – that might be useful in swaying opinions of stakeholders versus late analysis that is done 'just in time' to inform a decision.

PCT Board members expressed that our HIA as a useful, impartial tool in advising board members as to a decision to support the proposed reconfiguration. However, the timing of the report's presentation to the PCT Board was less than optimal for maximal effect; a strong request was made for this type of analysis to be available at the start of a consultation. Due to the late presentation of this assessment to the PCT Board its role was relegated to that of supportive in their decision-making rather than advisory.

Consideration should be given to the timing, depth and detail of analysis such as in this HIA and the time decision makers will have to study in detail. Having detailed analysis available at the start of public consultation might assist with framing contentious issues appropriately; though we stress that in this case, evidence alone may not change hearts and minds. Proposals to change maternity care have been particularly political in nature, with involvement of local and national politicians, including the Secretary of State [26]. This may be a reflection of a relatively large, well mobilised and well organised movement against such changes. In addition, differential perceptions of risk may be a factor, especially given that maternity care and childbirth display some of the 'fright factors' that reemphasise the need for very careful risk communication – focusing on absolute risks [27].

There is also a perceived need to be impartial in the analysis conducted – we view the role of the HIA in informing a decision based on impartial analysis; rather than providing supporting evidence with which to frame a pre judged conclusion. Although we set out to conduct an impartial analysis, we conducted this analysis from within the PCT, hence by definition we may have been susceptible to bias; we leave this for others to judge.

## Conclusion

Our analysis suggests that the proposed maternity service reconfiguration could improve antenatal and postnatal care. In turn, and in concert with a range of other services, the reconfiguration has the potential to reduce inequalities in maternal and infant health outcome in Kirklees. This is dependant on release of savings realised from a reconfiguration to ante natal services in the community. There are risks associated with Midwife Led Units. Some these might be mitigated by appropriate strategies. Recommendations from our HIA could ensure the delivery of maternity services in Kirklees that are in close keeping with the latest Government's view [28]. Conducting a Health Impact Assessment using this methodology is simple, efficient and can inform decision making processes. Careful consideration should be given to both the timing and the political context in which an analysis is undertaken.

## Competing interests

Both authors were on placement with (though not employed by) the Primary Care Trust at the time this health impact assessment was undertaken.

## Contributions of the authors

Both authors undertook the original study. GF wrote the first draft of this manuscript. SH redrafted. Both authors contributed equally to the publication.

## Pre-publication history

The pre-publication history for this paper can be accessed here:


